# La1: an evolutionarily conserved player in the *Arabidopsis* telomerase complex

**DOI:** 10.1007/s00299-026-03923-5

**Published:** 2026-08-06

**Authors:** Chinmay Phadke, Saundarya K. Mishra, Jiarui Song, Rebekah Holtsclaw, Claudia Castillo Gonzalez, Ishan Kundel, Edward M. Marcotte, Ophelia Papoulas, Dorothy E. Shippen

**Affiliations:** 1 Department of Biochemistry and Biophysics, Texas A&M University, College Station, TX 77843-2128 USA; 2 Department of Molecular Biosciences, The University of Texas at Austin, Austin, TX 78712 USA

**Keywords:** Ribonucleoprotein, RNP biogenesis, Dyskerin, RNAi knockdown, Telomerase RNA

## Abstract

**Key message:**

Quantitative mass spectrometry of *Arabidopsis* telomerase uncovered AtLa1, a homolog of ciliate and yeast proteins that promotes telomerase maturation. AtLa1 is essential for telomerase function in vivo, and in vitro it engages the same region of AtTR bound by AtNAP57, homologous to a telomerase accessory from mammals.

**Abstract:**

Striking divergence is evident in the biogenesis pathways and protein complements of telomerase from mammals and single-cell organisms. However, little is known about plant telomerase. Along with catalytic subunit TERT and templating RNA TR, we previously showed *Arabidopsis thaliana* telomerase is also associated with AtNAP57, a dyskerin homolog essential for mammalian telomerase biogenesis. Here we employ quantitative mass spectrometry (qMS) to uncover additional *Arabidopsis* telomerase constituents. We report AtLa1 as a new telomerase-associated protein. RNA-IP assays confirmed AtLa1 association with AtTR, while transient RNAi-mediated knockdown of AtLa1 strongly diminished telomerase activity, supporting a functional role for AtLa1 in telomere maintenance in vivo. In vitro binding studies revealed AtLa1 contacts AtTR via the UUU-3′OH and a plant-specific P1a-P1b-P4 three-way junction (TWJ). Notably, the TWJ is also required for AtNAP57 association with AtTR. However, this protein was not detected in our qMS experiment using overexpressed AtTERT and AtTR, perhaps because the purification scheme enriched for RNP assembly intermediates. La-related proteins serve as RNA chaperones and are associated with a wide variety of telomerase complexes. Therefore, we postulate that AtNAP57 and AtLa1 compete for AtTR or bind sequentially during telomerase biogenesis. Further exploration of *Arabidopsis* telomerase may offer novel insights into telomerase evolution and mechanisms of biogenesis.

**Supplementary Information:**

The online version contains supplementary material available at https://doi.org/10.1007/s00299-026-03923-5.

## Introduction

Telomeres consist of repetitive G-rich DNA sequences that cap chromosome ends. Telomeric DNA is synthesized by the telomerase reverse transcriptase, a ribonucleoprotein (RNP) enzyme that contains two core subunits: a catalytic protein, Telomerase Reverse Transcriptase (TERT), and an RNA moiety termed telomerase RNA (TR) that carries a telomeric DNA templating sequence. Although TERT and TR are sufficient to reconstitute telomerase enzyme activity in vitro, an array of accessory factors assemble with the core in vivo to facilitate RNP maturation as well as enzyme engagement and regulation at chromosome termini (Podlevsky and Chen 2016; Davis et al. 2023; Davis and Chakrabarti 2024). The protein composition of telomerase varies widely across eukaryotic lineages. Studies of the ciliate, yeast, and mammalian complexes reveal a multitude of protein–protein and RNA–protein interactions are required to form functional telomerase holoenzyme (Davis and Chakrabarti 2024). Some proteins stably associate with the core, while others interact transiently during RNP biogenesis and maturation.

Human telomerase harbors a 451-nt telomerase RNA (hTR) transcribed by RNA polymerase II (Feng et al. 1995; Cong et al. 1999). Biochemical and cryo-EM studies show the holoenzyme carries two copies of NHP2, NOP10, TCAB1, GAR1, and dyskerin, which together form a conserved H/ACA RNP scaffold essential for hTR stability and telomerase function (Chen et al. 2000; Venteicher et al. 2009; Nguyen et al. 2018; Ghanim et al. 2021). These proteins assist in RNA folding and facilitate its maturation and assembly into an active enzyme. They also promote proper localization of the telomerase complex within the nucleus, ensuring it reaches sites where telomere elongation occurs. Of particular note, dyskerin interacts with an H/ACA box near the 3’ terminus of hTR, contributing to RNA stability and maturation (Nguyen et al. 2018; Ghanim et al. 2021).

The budding yeast telomerase features a different set of protein components, including Est1, Est3, Pop1/6/7, yKu, and the Sm₇ ring complex. These accessory proteins support the stability, maturation, and activity of the *Saccharomyces cerevisiae* telomerase RNA (TLC1) and the telomerase enzyme complex in vivo. Est1 and Est3 are telomerase-specific regulatory proteins that recruit telomerase to telomeres and modulate its activity, with Est1 bridging the interaction between TLC1 and the telomere-binding protein Cdc13, and Est3 associating in a cell cycle-dependent manner (Lendvay et al. 1996; Chan et al. 2008; Chen et al. 2018). The Pop1/6/7 complex, shared with RNase P/MRP, binds to a conserved region of TLC1 and stabilizes the RNA, enhancing association with Est1 and Est2 (Lemieux et al. 2016; Garcia et al. 2020). The yKu heterodimer (yKu70/yKu80) binds to a stem–loop structure in TLC1 and is essential for its nuclear retention and telomeric localization, particularly during G_1_ (Fisher et al. 2004; Hass and Zappulla 2015; Garcia et al. 2020). The Sm₇ ring complex, a conserved group of RNA-binding proteins, associates with the 3′ end of TLC1 and plays a key role in its processing, stability and nuclear import (Seto et al. 1999; Vasianovich et al. 2020).

In *Schizosaccharomyces pombe*, a different set of proteins engages the telomerase RNA, including the La-related protein (LARP) Pof8, which promotes enzyme activity (Páez-Moscoso et al. 2018; Davis and Chakrabarti 2024). This finding is curious since La proteins typically associate with transcripts synthesized by RNA Pol III, but both the budding-and-fission yeast telomerase RNA molecules are generated by RNA Pol II. In addition, the majority of telomerase accessory factors are specific to yeast and not found in higher eukaryotes, emphasizing lineage-specific adaptations in telomerase biogenesis and regulation (Zappulla and Cech 2004; Lemieux et al. 2016).

The ciliate *Tetrahymena thermophila* possesses yet another structurally and compositionally distinct telomerase holoenzyme. TR is transcribed by RNA Pol III, and accessory proteins, such as p65, p75, p45, p19, p50, Teb1, Teb2, and Teb3, associate with the core enzyme (Jiang et al. 2013; Nguyen et al. 2018). Teb1 is a single-stranded DNA-binding protein that enhances telomerase processivity, functionally analogous (though not homologous) to human POT1 (Min and Collins 2010). Other subunits, such as p75 and p45, bridge the core RNP with the DNA substrate, supporting both recruitment and catalytic activity. Relevant to this study, p65, a LARP unique to *Tetrahymena* (Akiyama et al. 2012; Singh et al. 2012) promotes proper TR folding and stability.

Telomerases from the plant kingdom are less understood. Only recently was the bona fide TR identified (Fajkus et al. 2019; Song et al. 2019). Like other telomerase RNAs, the *Arabidopsis thaliana* telomerase RNA (AtTR) contains conserved core structural and functional domains, including a template region that guides telomere repeat synthesis and a pseudoknot domain that contributes to the structural integrity of TR and its catalytic function (Fajkus et al. 2019; Song et al. 2019; Štefanovie et al. 2024). AtTR also displays plant-specific features, including a unique three-way junction (TWJ) and a distinctive organization of hairpin loops and stem regions not found in vertebrate or yeast counterparts (Song et al. 2019; Štefanovie et al. 2024). These structural folds likely reflect plant-specific modes of RNA processing, stabilization and holoenzyme assembly.

A multitude of putative accessory proteins have been reported for *Arabidopsis* telomerase, but the functional relevance of most of these factors in telomere biology is unclear. For example, Telomere Repeat Binding (TRB) proteins from *A. thaliana* interact with both AtTERT and AtPOT1b, and are proposed to function in telomere protection and chromatin organization (Dvořáčková et al. 2015). TRB4 and TRB5 also serve as transcriptional activators of Polycomb Repressive Complex 2 (PRC2)-regulated genes, implying a connection between telomere biology and developmental processes such as flowering time control (Kusová et al. 2023). Several additional telomerase-associated proteins, including some involved in chromatin remodeling and transcriptional regulation, have been identified through yeast two-hybrid screening and co-immunoprecipitation assays. These factors include chromatin-associated proteins such as MSI1, a histone-binding protein linked to PRC2 function; NRP1 and NRP2, which are involved in nucleosome assembly; and VIM1, associated with DNA methylation (Schrumpfová et al. 2014; Fulnečková et al. 2021). More recent work reveals non-canonical functions for telomerase and telomere-related proteins in the stress response (Sharma et al. 2012; Barcenilla et al. 2023; Min et al. 2026), implying additional accessory factors remain to be determined. Although these findings underscore the multi-functionality of telomerase-associated proteins in coordinating chromosome end maintenance with broader epigenetic, metabolic, and developmental programs in plants, they provide limited mechanistic insights into fundamental aspects of telomerase RNP assembly, enzymatic function, and evolution.

We previously reported that *A. thaliana* telomerase engages AtPOT1a in vivo and in vitro (Surovtseva et al. 2007a, b; Arora et al. 2016). Protection of Telomeres (POT1) proteins in yeast and in mammals are stable components of the telomere and protect against a DNA damage response (Baumann and Cech 2001; Hockemeyer et al. 2005). By contrast, AtPOT1a accumulates at telomeres during S phase, and loss of AtPOT1a results in progressive telomere shortening, similar to loss of TERT (Surovtseva et al. 2007a, b). AtPOT1a is associated with enzymatically active telomerase, binding TERT independently of TR and stimulating repeat addition processivity (Surovtseva et al. 2007a, b; Renfrew et al. 2014; Song et al. 2021). The dyskerin homolog (AtNAP57) also interacts with *Arabidopsis* telomerase (Kannan et al. 2008; Song et al. 2021) as well as telomerase from *Allium cepa* (Fajkus et al. 2019). Analysis of an AtNAP57 loss-of-function mutant showed reduced telomerase activity and a shorter telomere length set point, indicating that dyskerin is essential for telomere length homeostasis in *Arabidopsis* (Kannan et al. 2008). Unlike AtPOT1a, AtNAP57 interacts with telomerase in an RNA-dependent manner (Song et al. 2021), similar to dyskerin in vertebrate telomerase (Mitchell et al. 1999; Garus and Autexier 2021). Notably, all the plant TR molecules characterized to date are transcribed by RNA Pol III (Fajkus et al. 2021). Therefore, the interaction of AtNAP57 with AtTR is unexpected. Biochemical studies revealed that AtNAP57 binds the TWJ within AtTR (Song et al. 2021), arguing that a plant-specific adaptation facilitates this protein–RNA interaction.

In this study, we employ quantitative mass spectrometry (qMS) to identify additional proteins that associate with *A. thaliana* telomerase. We present AtLa1 as a novel and functionally important telomerase-interacting protein for *Arabidopsis* and show that AtLa1 engages AtTR through the same domains as dyskerin. Our findings suggest that AtLa1 and dyskerin may be involved in a sequential assembly process during the biogenesis and maturation of *Arabidopsis* telomerase.

## Results

### qMS reveals a novel telomerase-interacting protein, AtLa1

To identify proteins associated with the *A. thaliana* telomerase core complex, we prepared a transgenic ‘super-telomerase’ line that overexpresses both AtTERT and AtTR by employing a binary vector carrying a 35S promoter-driven TwinStrep (TSII) tagged AtTERT and a U6 promoter-driven AtTR. Tagged-AtTERT was cloned using the genomic AtTERT sequence (5380 bp), including all introns with a TSII tag attached to the N-terminus, and the AtTR sequence (498 bp) was cloned along with the U6 promoter and terminator. This binary vector was transformed into *tert* null mutants. Two independent lines, 12-5 and 10-3, were identified which exhibited 2-fold and 40-fold higher telomerase activity, respectively, than the wild type (Barcenilla et al. 2023). Terminal Restriction Fragment (TRF) analysis indicated these lines complemented the *tert* null allele and exhibited slightly longer telomeres than wild type (Barcenilla et al. 2023).

**Fig. 1:**
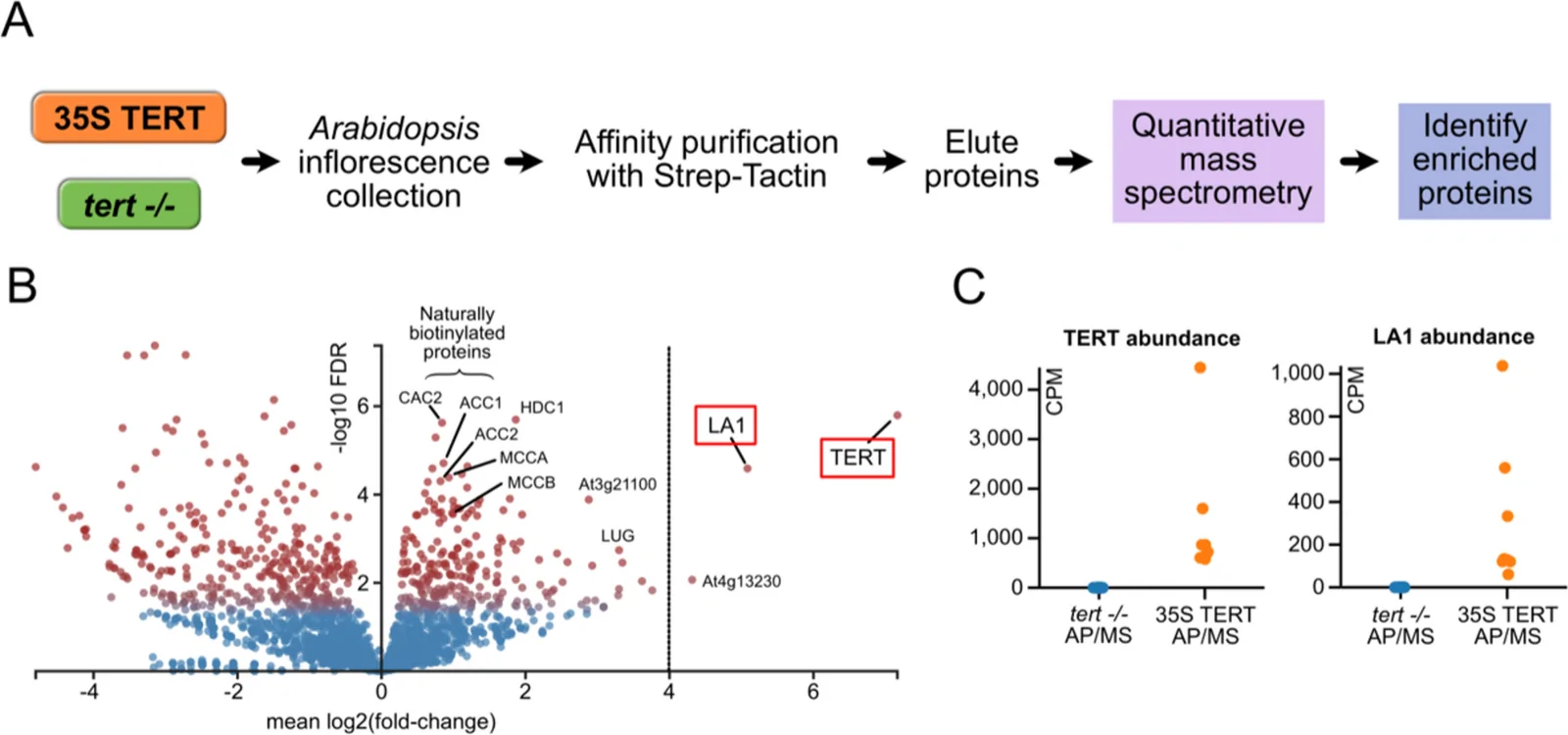
AtLa1 co-purifies with *A. thaliana* telomerase RNP. **A** Protocol for the extraction and affinity purification of telomerase RNP. **B** Volcano plot of protein fold-change (*x*-axis) and statistical significance (*y*-axis; *FDR* false discovery rate, as measured by EdgeR) in affinity purifications from 35S TERT plants relative to the untransformed control plants (*tert -/-*), showing that AtLa1 is the most highly enriched protein after TERT and hence likely involved in the telomerase RNP. AtTERT and AtLa1 are highlighted in red. **C** Mass spectrometry-measured protein abundances (in counts per million, *cpm*) for AtTERT and AtLa1, showing the high level of enrichment compared to *tert* null controls

We made several attempts to purify a tagged ‘native-telomerase’ so that normal stoichiometry and turnover conditions would be met by generating transgenic callus expressing TSII-tagged TERT from its native promoter. However, qMS experiments using these lines failed to identify any specific TERT-associated proteins, likely because of the very low abundance of native telomerase particles. Because of this issue, we decided to focus our purification efforts on the telomerase high activity line (line 10-3), extracting protein from its inflorescences as well as from *tert* mutant controls. TSII-tagged AtTERT was captured on strep-Tactin beads, the eluted proteins were concentrated and then subjected to qMS analysis (Fig. 1A).

Relative protein abundances in super-telomerase plants compared to the *tert* mutant plants were quantified from four biological replicates, using label-free protein quantification. Differentially enriched proteins were identified (see Methods) and are displayed in Fig. 1B as a volcano plot where both axes are log-transformed, enabling visualization of proteins statistically enriched in the affinity-purified samples. As expected, AtTERT was the most highly enriched protein in super-telomerase relative to the *tert* mutant samples, with an enrichment significantly greater than the naturally biotinylated proteins observed in this analysis, which might be expected to be at comparable levels in both the super-telomerase and *tert* mutant samples. Interestingly, the next most significantly enriched protein was the La-related RNA-binding protein AtLa1 (Fleurdépine et al. 2007), with a greater than 30-fold enrichment relative to the *tert* null control, which showed negligible background (Fig. 1C). Since La-related proteins have been detected in telomerase complexes from multiple model systems (Akiyama et al. 2012; Singh et al. 2012; Páez-Moscoso, et al., 2018; Davis and Chakrabarti 2024), we assessed the functional relevance of this protein in *Arabidopsis* telomere biology.

### AtLa1 is required for telomerase function in vivo

**Fig. 2:**
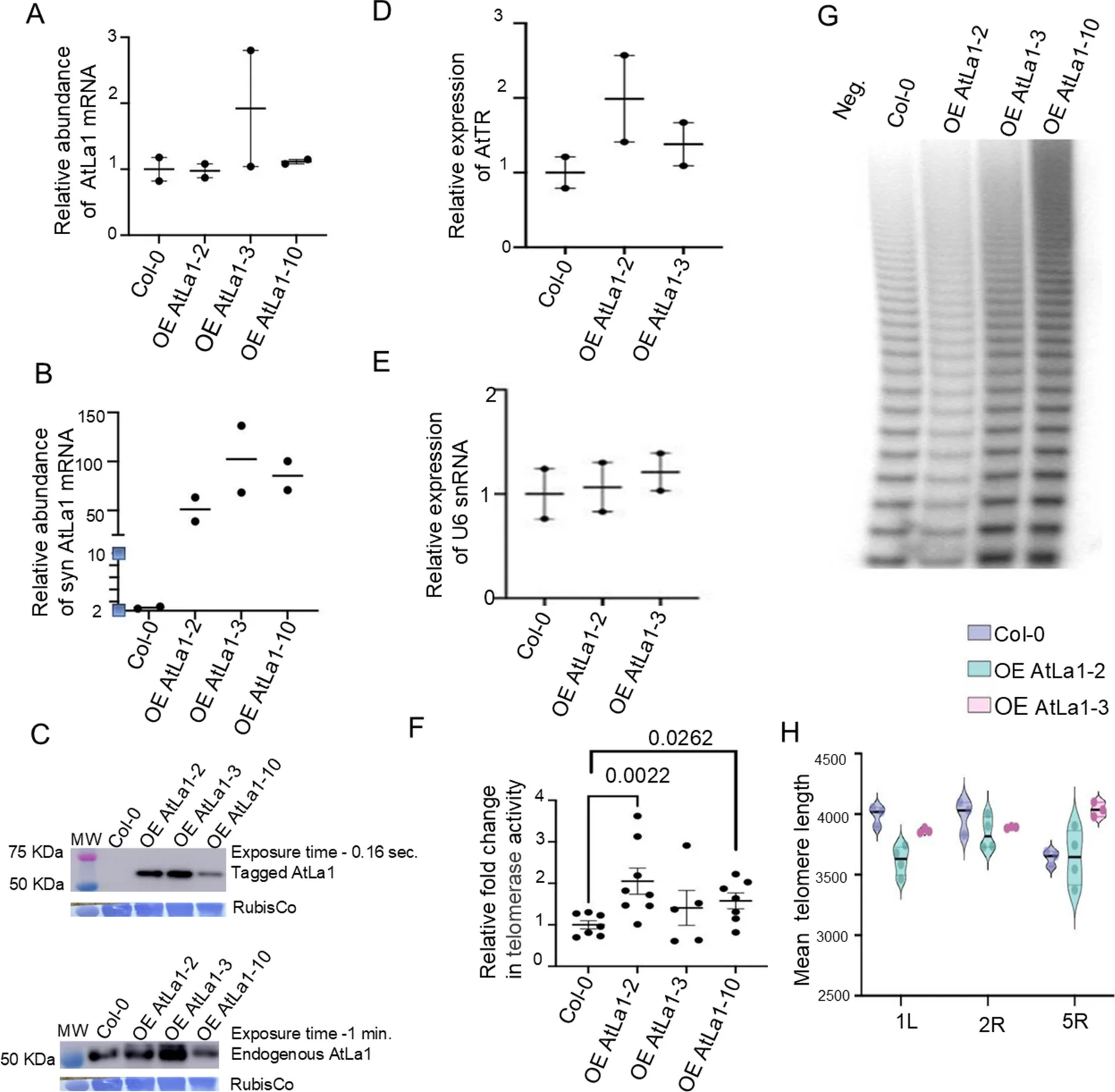
Over-expression of AtLa1 increases telomerase activity in vivo. Characterization of AtLa1 over-expression lines. **A** Results of quantitative PCR analysis for endogenous AtLa1 transcripts in wild type Col-0 and three AtLa1 over-expression lines. Data were normalized to *ACTIN2* levels. **B** Quantitative PCR results for AtLa1 syn transcripts in these same lines, data normalized to *ACTIN2*. **C** Western blotting results for tagged and endogenous AtLa1 protein expression in transformed lines OE AtLa1-2, OE AtLa1-3 and OE AtLa1-10 as compared to wild type Col-0. Blots were probed with α-AtLa1 primary antibody. RuBisCO served as a loading control. Molecular weight markers are (50–75 kDa) shown on the left of the blot. Quantitative PCR analysis of AtTR (**D**) and U6 snRNA (**E**) transcript abundance are shown. Data normalized with GAPDH. Quantitative Telomeric Repeat Amplification Protocol (qTRAP) (**F**) and conventional PAGE-resolved TRAP assay products (**G**) for lines over-expressing AtLa1. **H** Primer Extension Telomere Repeat Amplification (*PETRA*) results for mean length of telomeres on 1L, 2R and 5R chromosome ends with data analyzed by WALTER

AtLa1 is an RNA-binding protein (Maraia and Intine 2002; Wolin and Cedervall 2002) that recognizes the 3’ UUU of Pol III transcripts (Fleurdépine et al. 2007). Moreover, the canonical RNA recognition motifs (RRM) of AtLa1 are sufficient to restore La protein function in yeast, including U6 snRNA biogenesis, implying a conserved role in RNP assembly. La1 protein also participates in the maturation and stabilization of numerous noncoding RNAs including tRNA, U snRNA and U3 snoRNA (Cui et al. 2015). To investigate the biological relevance of AtLa1 for *Arabidopsis* telomerase, we used a previously characterized transgenic line of *A. thaliana* over-expressing tagged AtLa1 (35S:HA:AtLa1_syn_) (Cui et al. 2015). 14 progeny lines derived from this original line were characterized for AtLa1 expression by western blot using anti-HA-HRP to detect tagged AtLa1 (Fig. S1A, B). Quantitative PCR and western blotting verified over-expression of tagged AtLa1 in three lines (OE AtLa1-2, OE AtLa1-3 and OE AtLa1-10) relative to endogenous AtLa1 (Fig. 2A, B, C and S1C). Specifically, we detected 50- to 150-fold higher transcript levels and 8.9- to 9.5-fold higher protein expression in the 35S:HA:AtLa1_syn_ lines compared to the untransformed wild type Col-0 control. We confirmed that all three lines had a similar level of endogenous AtLa1 transcript and protein expression relative to wild type.

We monitored the steady-state level of AtTR transcripts in plants over-expressing AtLa1, reasoning that if AtLa1 is important for AtTR stability, AtTR levels might increase. We found a modest (1.5-fold) increase in AtTR, but no increase in U6 snRNA transcripts in the 35S:HA:AtLa1_syn_ lines compared to wildtype (Fig. 2D and E). We measured telomerase enzyme activity using conventional Telomere Repeat Amplification Protocol (TRAP) and quantitative TRAP (qTRAP) to check how AtLa1 over-expression affects telomerase activity. We observed a significant increase in telomerase activity in the transgenic lines (Fig. 2F and G), suggesting that AtLa1 over-expression promotes telomerase activity. Telomere length analysis using Primer Extension Telomere Repeat Amplification (PETRA) revealed no significant change when AtLa1 was over-expressed (Fig. 2H, S1D, E, F), consistent with previous studies showing that elevated levels of telomerase enzyme activity do not lead to telomere elongation in *Arabidopsis* (Barcenilla et al. 2023).

**Fig. 3:**
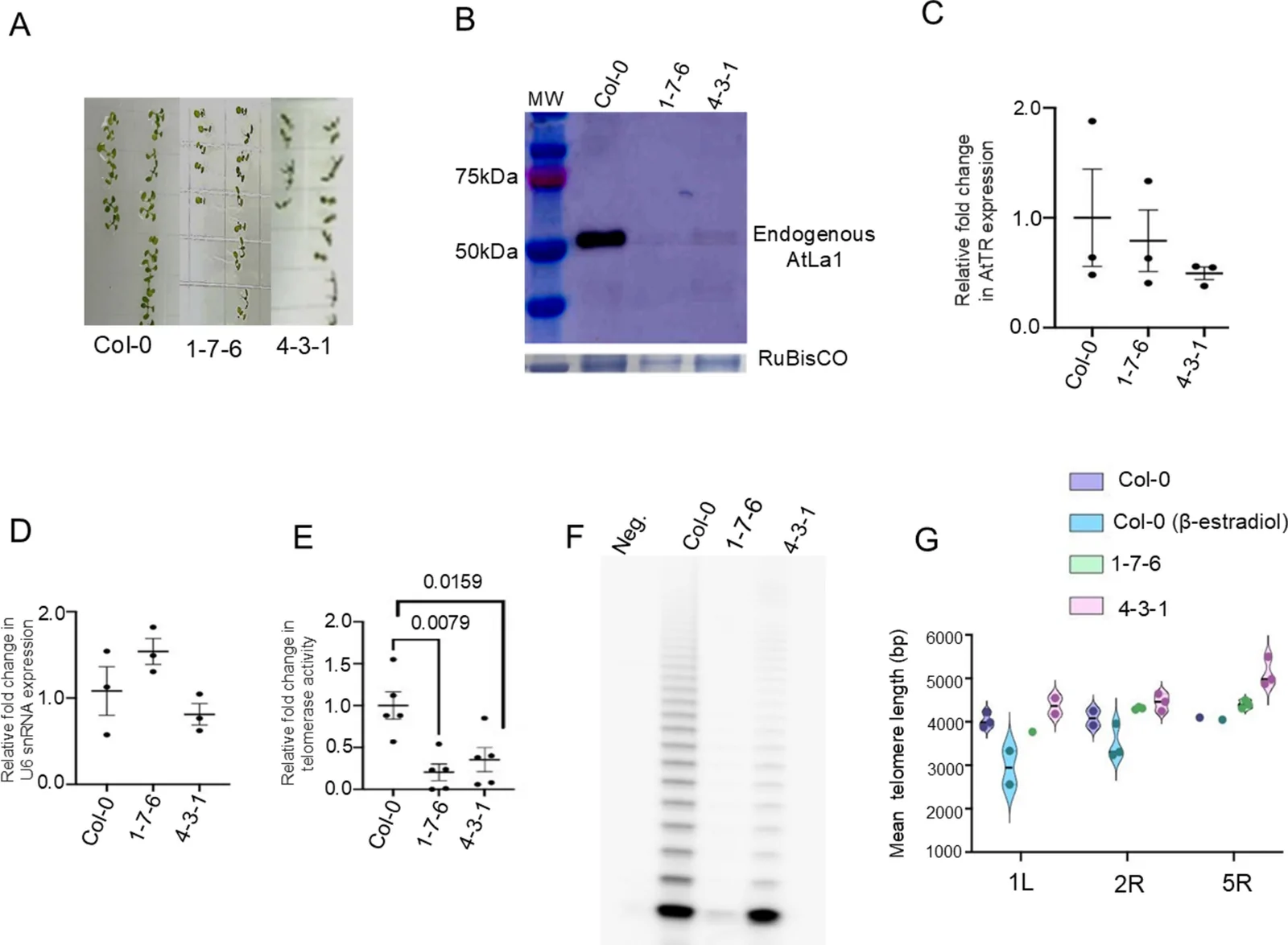
Knock-down of AtLa1 reduces telomerase activity. **A** Reduced growth observed in seven-day-old seedlings transformed with an inducible RNAi construct (lines 1-7-6 and 4-3-1) relative to the wild type (Col-0). Seedlings were grown in MS plates supplemented with 25 µM β-estradiol. **B** Characterization of inducible RNAi knockdown lines by western blot analysis (blot probed with anti-AtLa1 primary antibody). RuBisCO served as a loading control. Molecular weight markers are (50–75 kDa) shown on the left of the blot. **C** and **D** Results of qPCR analysis for AtTR and U6 snRNA abundance in knockdown lines and Col-0 controls. Data were normalized with GAPDH. **E** and **F** Results of qTRAP and conventional TRAP for AtLa1 inducible RNAi knockdown lines (1-7-6 and 4-3-1) as compared to wild type Col-0. **G** Average telomere length analyzed in untreated Col-0, β-estradiol-treated Col-0 and RNAi knockdown lines 1-7-6 and 4-3-1. Data analyzed by WALTER

AtLa1 is essential for embryonic development (Fleurdépine et al. 2007), maintaining stem cell homeostasis by promoting translation of WUSCHEL (WUS) mRNA (Cui et al. 2015). Consequently, depletion of AtLa1 likely triggers a variety of pleiotropic effects. To mitigate such effects, we examined the impact of AtLa1 depletion by creating a transiently inducible RNAi knock-down line. Specifically, we generated homozygous (T3) lines containing a β-estradiol-inducible RNAi knock-down construct (pER8-hpRNAiAtLa1) to control AtLa1 expression post-embryonically. These lines were considered in parallel with wild type Col-0 and were plated on 0.5 MS supplemented with 25 μM/ml β-estradiol (Fig. 3A). Western blotting with seven-day-old seedlings showed a 10- to 60-fold reduction in AtLa1 protein expression in 4-3-1 and 1-7-6 lines, respectively, as compared to the control (Fig. 3B).

We asked how AtLa1 depletion impacted AtTR and U6 snRNA accumulation and found no significant decrease in either RNA relative to the GAPDH control (Fig. 3C and D). Telomerase assays were then conducted to determine the effect of AtLa1 inhibition on enzyme activity. Unexpectedly, we observed a decrease in telomerase activity in the 25 μM/ml β-estradiol-treated wild type Col-0 control compared to untreated plants (Fig. S2A). Therefore, for subsequent experiments, we supplemented the media of both RNAi transgenic lines and untransformed Col-0 controls with 25 μM/ml β-estradiol (Fig. 3A). Telomerase activity measured by qTRAP on seven-day-old β-estradiol-treated seedlings showed a 6-fold decrease in activity in the 1-7-6 line and a 4-fold decrease in the 4-3-1 line, relative to the wild type Col-0 control (Fig. 3E). We also examined telomerase activity using the conventional gel-based TRAP assay. Relative to the Col-0 control, enzyme activity was decreased in both lines, but more so in 1-7-6 (Fig. 3F), consistent with the qTRAP findings (Fig. 3E). Finally, PETRA was performed on DNA extracted from seven-day-old seedlings post AtLa1 knockdown to assess telomere length. Examination of telomeres on three independent chromosome arms revealed no significant change compared to the non-transformed control lines (Fig. 3G, S2B, C and D). Nevertheless, the striking reduction in telomerase enzyme activity when AtLa1 levels were decreased argues that AtLa1 is required for telomerase function in vivo.

### AtLa1 does not enhance the enzyme activity of telomerase RNP particles reconstituted in vitro

**Fig. 4:**
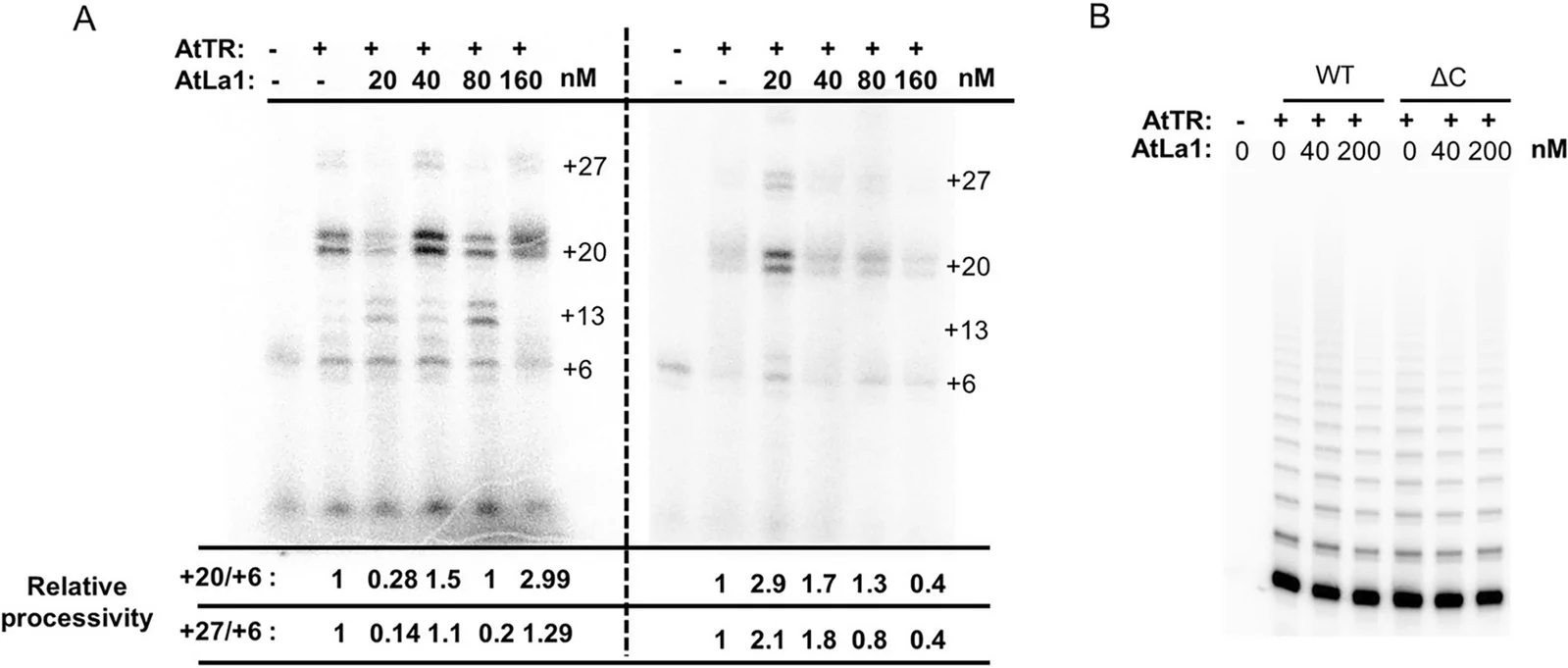
Effect of AtLa1 on *Arabidopsis* telomerase activity in vitro. **A** Results of two independent direct telomerase primer extension assays for telomerase reconstituted in vitro (RRL-expressed AtTERT and AtTR) in the presence of increasing concentrations of *E. coli* expressed AtLa1 (20–160 nM). Dashed line marks the two different reconstitution experiments. AtLa1 did not reproducibly enhance relative repeat addition processivity, measured as the ratio of signal intensity between longer extension products (+ 27) and shorter products (+ 6). **B** TRAP assay results for telomerase reconstitution reactions containing increasing concentrations of AtLa1_WT_ and AtLa1_ΔC_

To investigate how AtLa1 influences telomerase activity, we asked if enzyme activity was enhanced when *E. coli* expressed and purified AtLa1 was included in an in vitro telomerase reconstitution experiment containing Rabbit Reticulocyte (RRL) expressed AtTERT and AtTR (Song et al. 2019, 2021). We assessed telomerase activity by employing a direct primer elongation assay (without PCR amplification). We observed no changes in enzyme activity that correlated with AtLa1 protein concentration (Fig. 4A). In addition, when we measured the number of telomere repeats incorporated in the presence and absence of AtLa1, we found no difference (Fig. 4A), arguing that AtLa1 does not stimulate overall enzyme activity or repeat addition processivity under these conditions. Similar results were obtained when the reconstitution reactions, supplemented with either full-length AtLa1 or the truncated version, AtLa1_ΔC_, which lacks the ability to bind AtTR (see below), were evaluated by the PCR-based TRAP assay (Fig. 4B). We note that a strikingly different result was observed when recombinant AtNAP57 was subjected to the same in vitro reconstitution protocol. In this case, a clear enhancement of both enzyme activity and repeat addition processivity was detected (Song et al. 2021). The current data suggest that AtLa1 does not promote telomerase enzyme activity in the same manner as dyskerin, at least under these in vitro reaction conditions.

### AtLa1 interacts with AtTR

**Fig. 5:**
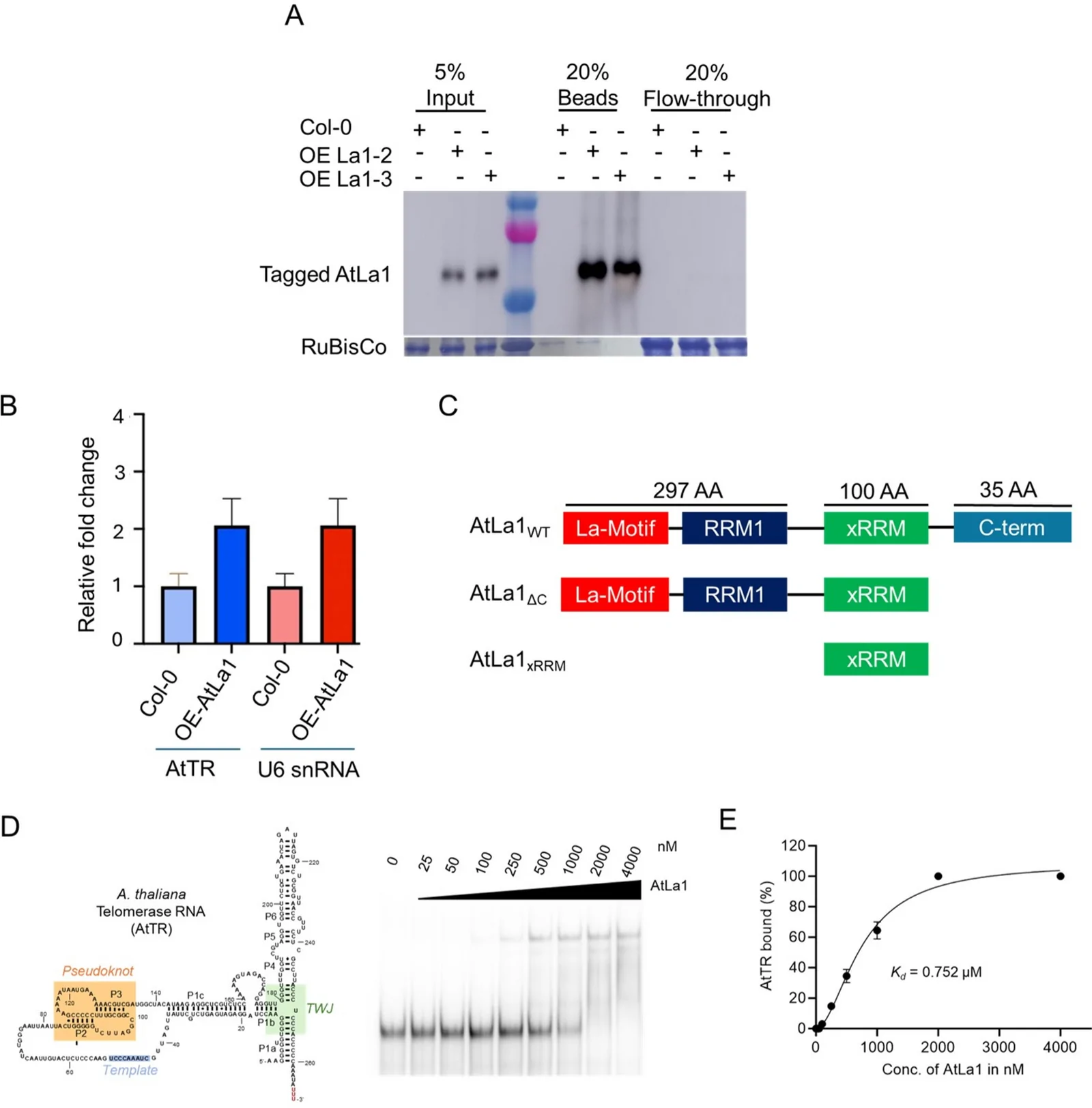
AtLa1_WT_ binds full-length AtTR in vitro. RNA immunoprecipitation (RIP) results obtained for 35S:HA:AtLa1_syn_ and Col-0 controls. Affinity purified samples were subjected to western blotting with anti-HA-HRP (**A**). RuBisCo served as a loading control. qPCR results are shown in (**B**). **C** Domain organization of AtLa1_WT_ (433 amino acids) and its truncations used in EMSA. Radiolabeled AtTR was incubated with increasing concentrations of recombinant AtLa1 protein (0–4 μM). **D** Left, predicted secondary structure of *A. thaliana* telomerase RNA (*AtTR*), highlighting the pseudoknot (*PK*), template domain, and plant-specific three-way junction (*TWJ*). Right, EMSA showing binding of increasing concentrations (0–4 µM) of purified recombinant AtLa1_WT_ to in vitro-transcribed, PAGE-purified, radiolabeled AtTR. **E** Quantification of EMSA data reveals that AtLa1_WT_ binds AtTR with a dissociation constant (*K*_*d*_) of 0.752 µM

Given the capacity of AtLa1 to bind RNA (Maraia and Intine 2002; Wolin and Cedervall 2002), we performed RNA-IP to determine if AtLa1 is associated with AtTR in vivo, using 35S:HA:AtLa1_syn_. Western blotting confirmed that HA-tagged AtLa1 was enriched when extracts from plants expressing 35S:HA:AtLa1_syn_ were subjected to affinity purification (Fig. 5A). We used qPCR to determine if AtTR was likewise enriched in this purification. The level of AtTR and U6 snRNA abundance increased by 2- and 2.5-fold in AtLa1 over-expression lines as compared to untransformed wild Col-0. In this study, U6 snRNA was employed as a positive control since AtLa1 has been shown to associate with this RNA (Fleurdépine et al. 2007) (Fig. 5B). These findings indicate that AtLa1 is associated with *Arabidopsis* telomerase complexes containing AtTR in vivo.

We next used Electrophoretic Mobility Shift Assays (EMSA) to investigate whether AtLa1 directly binds AtTR. We first tested AtTR interaction using full-length AtLa1 (AtLa1_WT_) (433 amino acids: 6xHis-SUMO-AtLa1_WT_) (Fig. 5C). Full-length wild type AtTR was prepared by in vitro transcription, and the RNA was purified by 6% denaturing PAGE (Fig. S3A). AtLa1_WT_ was expressed and purified from *E. coli* (Rosetta cells) (Fig. S3B). Fractions 13 and 14 with a purity of ~ 95% were used for the EMSA assay. EMSA data indicated that AtLa1_WT_ binds AtTR in vitro with a dissociation constant (*K*_*d*_) of 0.752 µM (Fig. 5D and E). The observed sub-micromolar dissociation constant indicates a moderate affinity interaction under these in vitro conditions.

AtLa1 contains a canonical La domain, followed by RNA Recognition Motif 1 (RRM1), a poorly characterized RRM2 (also referred to as xRRM) and a short C-terminal region (Fig. 5C) (Bousquet-Antonelli and Deragon 2009). To dissect the contribution of individual domains in AtLa1 toward AtTR binding, we expressed and purified two truncation constructs in *E. coli*: 6xHis-SUMO–AtLa1_ΔC_, lacking the 36-residue C-terminal region; and 6xHis-SUMO–AtLa1_RRM2_, a 100-residue construct comprising only RRM2 (Fig. S3B and C). EMSA analyses did not reveal discrete shifted complexes for either truncation construct (Fig. S4A and B). However, in Fig. S4B, a substantial reduction in free RNA probes was observed in the presence of AtLa1_RRM_ without formation of a defined mobility shift. Such behavior may reflect altered RNA conformations or dynamic protein–RNA assemblies, raising the possibility that AtLa1 influences RNA structural states in addition to forming stable binary complexes. These findings also indicate that the canonical La domain, RRM1, as well as the C-terminal region of AtLa1 is needed for AtTR interaction.

### AtLa1 binds AtTR via a plant-specific TWJ and the UUU-3’OH

**Fig. 6:**
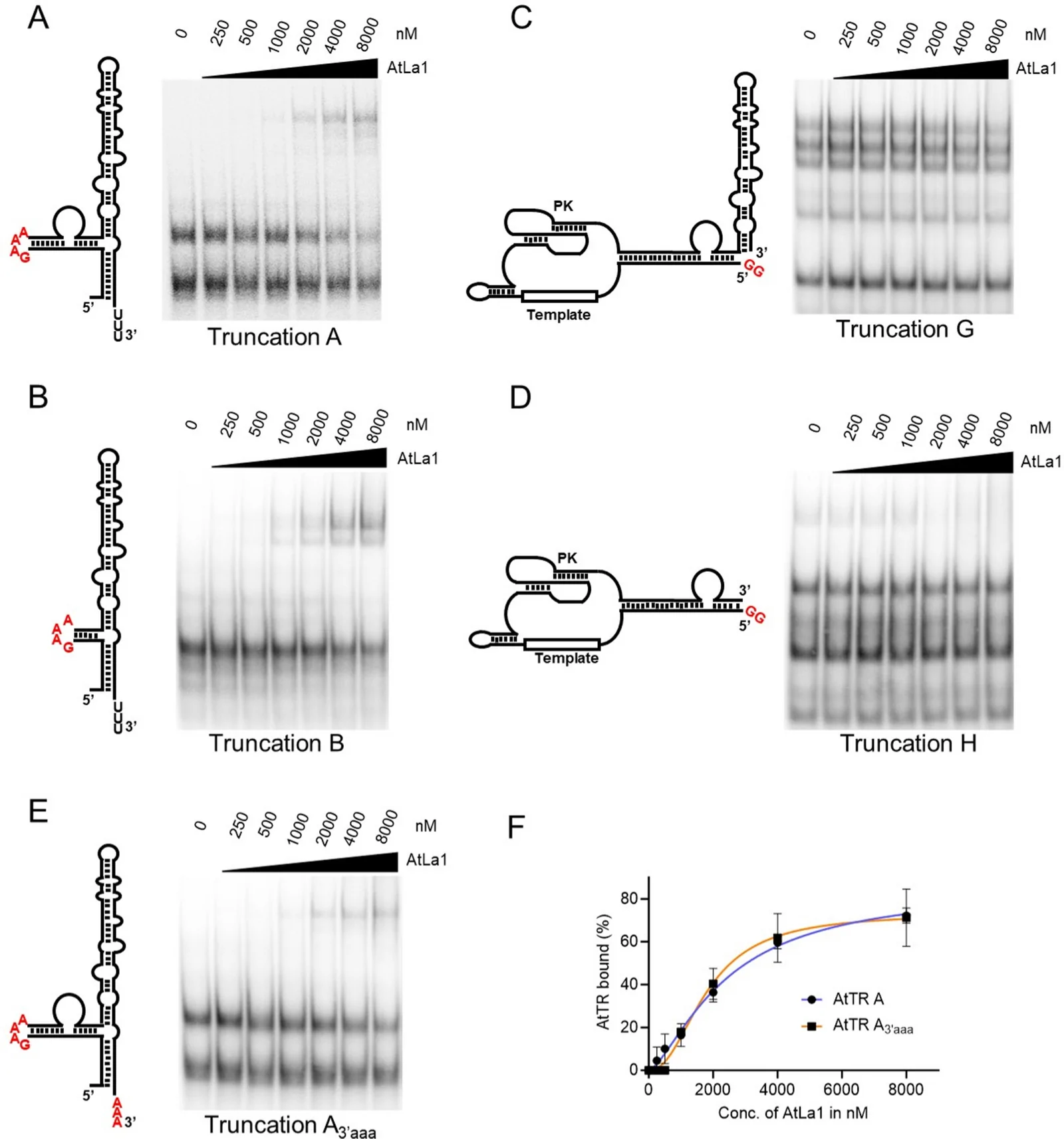
AtLa1 binds *Arabidopsis* telomerase RNA through the plant-specific three-way junction (*TWJ*). EMSA analyzing the interaction of AtLa1_WT_ with AtTR truncation constructs. Radiolabeled RNAs were incubated with increasing concentrations of AtLa1_WT_ (0–2 μM). **A** Truncation A, lacking the pseudoknot (*PK*) and template region, bound AtLa1 with ~ 3-fold reduced affinity (*K*_*d,app*_ = 2.40 μM) compared to full-length AtTR. **B** Truncation B, which additionally lacks the bulge between P1c and P1b, bound AtLa1 with ~ 2-fold reduced affinity (*K*_*d,app*_ = 1.62 µM). **C**, **D** Truncations G and H, which retain the PK and template domains but lack the 3′ stem P1a (H additionally lacks P4–P6), failed to bind AtLa1, indicating that the TWJ is essential for productive interaction. **E** AtLa1 bound A_3’aaa_ with a dissociation constant (*K*_*d,app*_) of 1.80 μM, indicating that AtLa1 interaction is retained despite loss of the UUU-3’OH motif. **F** Quantification of EMSA data comparing truncation A and A_3’aaa_

**Fig. 7:**
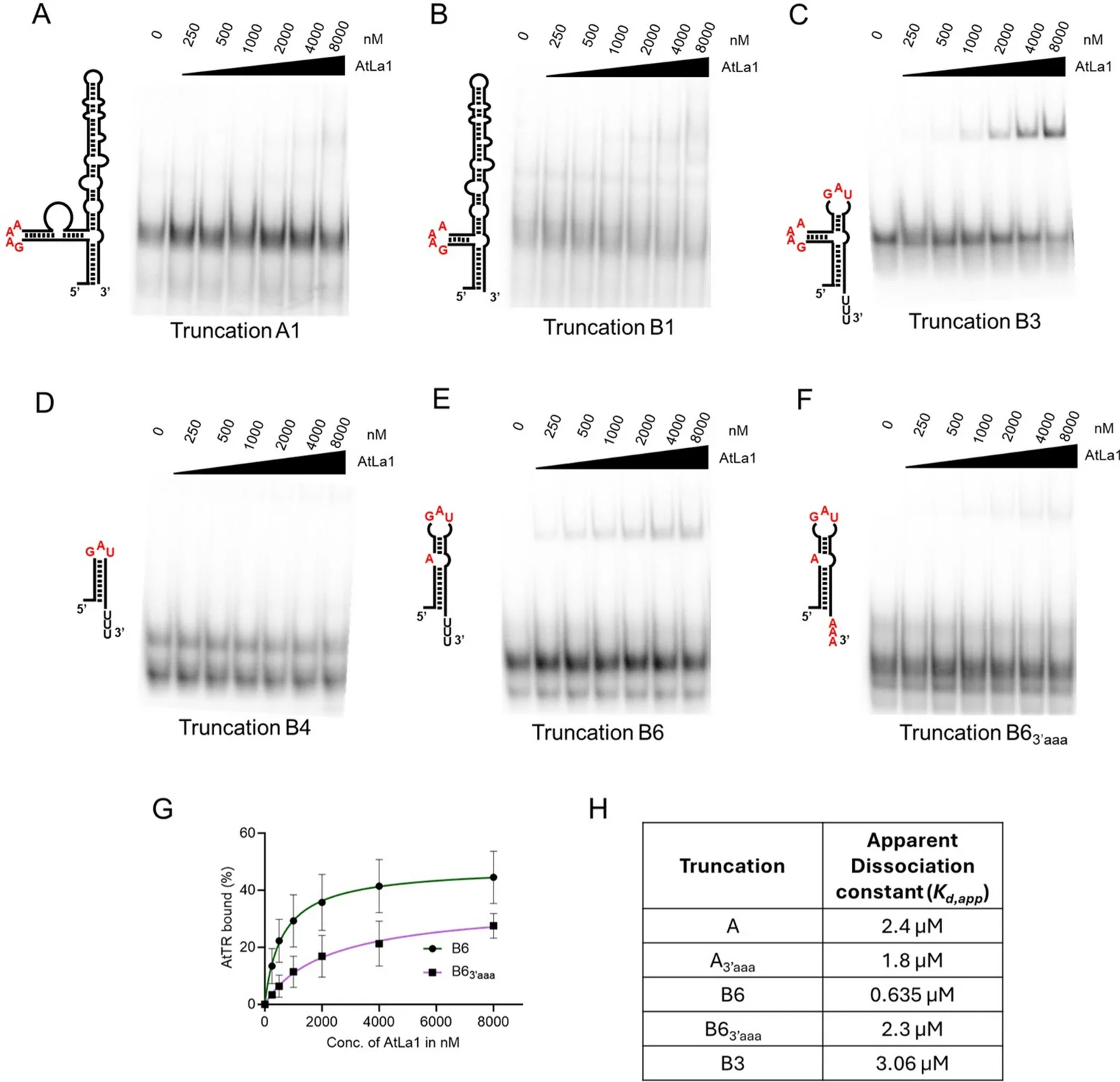
AtLa1 selectively binds to the UUU-3’OH. EMSA analyzing binding of AtLa1 to AtTR fragments. Radiolabeled RNA constructs were incubated with increasing concentrations of recombinant AtLa1 protein (0–2 μM). **A**, **B** Truncations A1 (**A**) and B1 (**B**) exhibited only weak binding that was not quantifiable. **C** Truncation B3 displayed moderate binding to AtTR. **D** Truncation B4, lacking the TWJ but retaining the UUU-3′OH, showed no binding, highlighting the requirement of the TWJ for interaction. **E** Truncation B6 showed the strongest binding affinity, with a dissociation constant (*K*_*d,app*_) of 0.635 μM. **F** Replacement of the UUU-3′OH with AAA-3′OH in B6 (B6_3’aaa_) reduced the affinity by ~ 4-fold, demonstrating the requirement of the U-rich tail for AtLa1 binding. **G** Binding analysis of B6 and B6_3’aaa_. **H** Summary of AtTR truncations and their binding to AtLa1

To pinpoint the binding site of AtLa1 on AtTR, we prepared several AtTR truncation constructs (Figs. 6 and 7) and studied their binding with AtLa1_WT_. We first asked if the core structural and functional domains within AtTR are necessary for AtLa1 binding. Truncations A and B lack the pseudoknot (PK) and template region, while truncation B also lacks the bulge between P1c and P1b. AtLa1_WT_ retained binding to truncations A and B, but with reduced affinity compared with full-length AtTR. Binding was approximately 3-fold weaker for truncation A (*K*_*d,app*_ = 2.40 µM) and 2-fold weaker for truncation B (*K*_*d,app*_ = 1.62 µM) (Fig. 6A and B). Thus, the PK and template domains of AtTR are not essential for AtLa1 binding. To further investigate how these regions contribute to AtLa1 engagement, we prepared truncations G and H, which retain PK and template regions but lack the 3’ stem P1a. Truncation H also lacks P4, P5, and P6. Interestingly, no binding was detected for truncations G or H, suggesting that some features of the 3’ stem region are needed for interaction with AtLa1_WT_ (Fig. 6C and D).

One unique feature of AtTR is a plant-specific Three-Way Junction (TWJ) composed of the P1a stem, bearing a small stretch of Single-Stranded RNA (SSR) consisting of a poly U stretch at RNA 3’ terminus (UUU-3’OH), the P1b stem, and the P4 stem, which appears to correspond to eCR4/5 of the human telomerase RNA (Song et al. 2019). We previously showed that the TWJ is necessary for AtNAP57 binding (Song et al. 2021). Notably, truncations A and B share the single-stranded part of the 3’ stem, which may serve as the site of interaction for AtLa1_WT_. Since La proteins are known to bind the UUU-3′OH of Pol III transcripts (Stefano 1984; Gottlieb and Steitz 1989; Wolin and Cedervall 2002), we replaced the UUU-3’OH on truncation A with AAA-3’OH (A_3’aaa_) to determine if another domain in the RNA is contacted by AtLa1. EMSA revealed that AtLa1_WT_ interacted with A_3’aaa_ with a dissociation constant (*K*_*d,app*_) of 1.80 µM (Fig. 6E). Quantification of EMSA data comparing truncation A and A_3’aaa_ was performed by calculating the fraction of bound RNA (shifted signal divided by total signal, bound + free). Although shifted band intensities appeared similar between constructs (Fig. 6A, E), differences in free RNA levels contributed to the calculated binding fractions (Fig. 6F). These analyses indicate that AtLa1 is not entirely dependent on the UUU-3’OH residues for interaction with AtTR and likely engages additional structural elements within the TWJ.

We then asked if the 3’ SSR is necessary for AtLa1 binding using truncations A1 and B1, which are similar to truncations A and B, but lack the 3’ SSR (Fig. 7A and B). EMSA revealed very weak binding insufficient to allow quantification, and no dissociation constant could be derived using ImageJ. To further refine the AtLa1-binding site, we deleted P5 and P6 from truncation B to prepare truncation B3. Additionally, we generated truncation B4 which only has 3’ stem P1a which includes a 3’ SSR region. We found that AtLa1 retains interaction with the B3 truncation with a dissociation constant (*K*_*d,app*_) of 3.06 µM (Fig. 7C). However, binding to B4 was strongly reduced relative to other constructs (Fig. 7D). Only faint complexes were detectable upon contrast adjustment, suggesting that interaction is markedly weakened but not entirely eliminated. Strikingly, removal of the P1b stem on B3 to generate truncation B6 resulted in the strongest binding affinity with a *K*_*d,app*_ of 0.635 µM (Fig. 7E). When we replaced the UUU-3’OH of B6 with AAA-3’OH to create construct B6_3’aaa_, there was a 4-fold decrease in the affinity (*K*_*d,app*_ = 2.356 µM) (Fig. 7F and G). Quantitative comparison of dissociation constants across truncations (Fig. 7H) highlights the enhanced affinity of B6 and the reduced binding upon disruption of either the TWJ architecture or the UUU-3′OH, underscoring the cooperative contribution of these elements to AtLa1 recognition. These results support a model in which AtLa1 engages structural elements within the TWJ in combination with the UUU-3’OH of AtTR, with both features contributing to binding. Taken together, our data demonstrate that AtLa1 engages AtTR, a core component of the *Arabidopsis* telomerase in vivo and in vitro.

## Discussion

Telomerases display a remarkable diversity of molecular strategies to regulate biogenesis, maturation, and assembly, and to ensure precise recruitment and engagement of the enzyme at chromosome termini. A wide variety of telomerase accessory factors emerged to facilitate key steps in telomerase regulation, including transcriptional control, stabilization and processing of TR, chaperoning of protein subunits, and coordination with cell cycle and DNA damage response pathways to restrict telomerase action to appropriate cellular contexts (Buchkovich and Greider 1996; Cifuentes-Rojas and Shippen 2012; Liu et al. 2024). Because comparatively little is known about telomerase in the plant kingdom, we employed an unbiased qMS approach to investigate whether RNP assembly and regulatory mechanisms similar or divergent to other eukaryotic lineages exist for *Arabidopsis* telomerase.

The most significantly enriched protein in our MS experiment, besides tagged TERT, was AtLa1. La-related proteins (LARPs) have previously been associated with ciliate, yeast, and mammalian telomerases (Aigner et al. 2000; Collopy et al. 2018; Kao et al. 2024). *Tetrahymena* p65 (a LARP7 family protein) induces structural changes in TR that promote RNP assembly (Aigner et al. 2000; O’Connor and Collins 2006). Similarly, in humans and yeast, LARP7 is important for telomerase enzyme activity and telomere length maintenance (Bousquet-Antonelli and Deragon 2009; Collopy et al. 2018; Kao et al. 2024). Interestingly, no LARP7 homologs have been identified in *Arabidopsis* (Xiang et al. 2024). However, the genuine La protein AtLa1, bearing the defining “La module,” a canonical La motif followed by an RNA Recognition Motif (RRM1), binds plant RNAs bearing a UUU-3’OH terminus, including U6 snRNA and pre-tRNA (Fleurdépine et al. 2007).

In conjunction with our qMS data, we also provide biochemical and genetic evidence that AtLa1 assembles with *Arabidopsis* telomerase. First, RNA-IP experiments confirmed that AtLa1 is associated with AtTR in vivo*,* while EMSA experiments demonstrated that AtLa1 directly binds AtTR in vitro. Second, analysis of transgenic plants over-expressing AtLa1 revealed increased telomerase activity, and transient knockdown of AtLa1 led to substantially decreased telomerase activity. These changes in telomerase activity are consistent with a critical role for this protein in facilitating telomerase function in vivo.

Interestingly, despite alterations in telomerase activity, we did not detect concomitant changes in telomere length. We did not observe shortened telomeres when telomerase activity was decreased in response to AtLa1 inhibition. We note that telomeres in *Arabidopsis* mutants null for *AtTERT* or *AtPOT1a* shorten by only 200–500 bp during the entire 6-week life cycle (Riha et al. 2001; Surovtseva et al. 2007a, b). Therefore, the brief duration of AtLa1 inhibition, only seven days, was likely insufficient to reveal telomere shortening using standard methodology. We also failed to observe telomere lengthening when telomerase activity levels were elevated in response to AtLa1 over-expression. Although increased telomerase activity drives telomere elongation in mammalian cells (Cristofari and Lingner 2006), this is not the case for *Arabidopsis*. In settings where telomerase activity is increased genetically or in response to oxidative stress, telomeres do not elongate (Ren et al. 2004; Barcenilla et al. 2023). Thus, telomere length is tightly regulated in *Arabidopsis* and excess telomerase is not employed for telomere elongation.

Our data indicate that AtLa1 directly associates with AtTR. A recent, independent biochemical study, published while this manuscript was in preparation, provides further evidence that AtLa1 binds AtTR (Jenner et al. 2026). Using microscale thermophoresis (MST), Jenner et al. observed a biphasic interaction between AtTR and AtLa1, with an initial high-affinity phase (*K*_*d*_ ≈ 5 ± 1 nM) associated with protein-induced fluorescence quenching and a lower-affinity phase (*K*_*d*_ ≈ 1.9 ± 0.2 µM) linked to larger RNA conformational perturbations. Thermophoresis measurements monitoring complex formation similarly yielded a high-affinity interaction (*K*_*d*_ ≈ 20 ± 10 nM). Our EMSA measurements revealed an affinity of *K*_*d*_ = 0.75 µM, close to the lower-affinity phase reported in the MST analysis, suggesting that our assay may have captured a later or more stable binding state. Together, these observations provide complementary insights into the AtLa1–AtTR interaction and support a model in which AtLa1 binding involves initial RNA recognition followed by structural remodeling of AtTR. Finally, we note the apparent affinity of AtLa1 for AtTR is lower than that reported for telomerase RNA-binding proteins such as p65 in *Tetrahymena* or Pof8 in fission yeast, which bind their cognate RNAs with affinities of ~ 25 nM and ~ 30–50 nM, respectively (Akiyama et al. 2012; Singh et al. 2012). However, the binding affinity we determined does not necessarily reflect the strength or stability of the AtLa1-AtTR interaction in vivo. Within the cellular context, additional telomerase-associated factors, local concentration effects within subnuclear compartments, RNA folding dynamics, and potential multivalent interactions may enhance effective binding and stabilize the complex beyond what is observed in a minimal in vitro system (O’Connor and Collins 2006; Stone et al. 2007).

Intriguingly, we found that AtLa1 recognizes the UUU-3’OH terminus of AtTR and the TWJ element unique to the plant telomerase RNA. Unlike canonical substrate RNAs which predominantly rely on the UUU-3’OH for AtLa1 recognition, the UUU-3’OH of AtTR seems dispensable for AtLa1 binding and can be compensated by the presence of the TWJ. Strikingly, AtNAP57 (dyskerin) requires the same sites on AtTR for binding (Song et al. 2021), prompting questions about the composition and timing of accessory factor association during telomerase biogenesis and maturation in *Arabidopsis*. Our qMS dataset did not contain several previously reported telomerase-associated proteins, including dyskerin, AtPOT1a, TRB proteins, and NAP1 (Kannan et al. 2008; Surovtseva et al. 2007a, b; Schrumpfová et al. 2014; Fulnečková et al. 2021). Peptides for NAP1 (Fulnečková et al. 2021) were detected in our dataset, but at statistically insignificant levels (Supplementary Table 1). Therefore, NAP1 was not included in the Fig. 1B volcano plot. The absence of proteins previously associated with *A. thaliana* telomerase may reflect both the dynamic nature of telomerase RNP assembly and the biochemical conditions used for our affinity purification.

Telomerase assembly is dynamic, and distinct complexes have been reported at different stages of biogenesis or maturation. For example, *Tetrahymena*, RNP assembly proceeds hierarchically, where La-family protein p65 first binds telomerase RNA and remodels its structure to then facilitate recruitment of TERT and additional holoenzyme components (O’Connor and Collins 2006; Berman et al. 2010; Singh et al. 2012). Accordingly, biochemical purifications recover telomerase complexes containing different subsets of accessory proteins, suggesting heterogeneous RNP particles that represent stepwise assembly or maturation states (Witkin and Collins 2004). In addition, our affinity purification employed relatively stringent extraction and wash conditions (high-salt and detergent-containing lysis buffer: 150 mM KGlu, 0.1% Triton X-100) designed to solubilize nuclear matrix complexes thoroughly, which may have preferentially preserved stable interactions while destabilizing more transient or lower-affinity associations with mature accessory factors. Thus, the absence of significant enrichment for dyskerin, POT1a, TRBs, and other reported components should not be interpreted as evidence that these proteins are absent from the mature *Arabidopsis* telomerase holoenzyme, but rather that our purification likely captured a subset of telomerase RNP complexes enriched for stable interactions involving AtLa1 and TERT.

We postulate our qMS experiments enriched for partially assembled, inactive complexes that have not yet undergone complete maturation. Because only a few particles of active telomerase are present in cells, over-expressed AtTERT and AtTR may preferentially associate with factors that bind early in RNP biogenesis. The absence of dyskerin or any of the other previously characterized telomerase accessory factors does not preclude their presence in a mature, fully active telomerase complex. We propose that AtLa1 transiently associates with AtTR during early biogenesis to enhance RNP assembly before being displaced by other factors necessary for maturation and robust repeat addition processivity (Kannan et al. 2008; Renfrew et al. 2014; Arora et al. 2016; Song et al. 2021).The capacity of AtLa1 to bind AtTR, particularly to the TWJ and the 3′ U-rich single-stranded region, suggests a potential role in stabilizing immature AtTR transcripts or protecting their 3′ ends, analogous to the protective function of other La proteins on RNA Pol III products (Maraia and Intine 2002; Naeeni et al. 2012). While our data indicate that AtLa1 is not sufficient to promote enzymatic activity in in vitro reconstitution assays, our inducible AtLa1 knockdown lines consistently showed reduced telomerase activity. This decrease did not correlate with a decrease in the steady-state level of AtTR. Importantly, however, the La motif and RRM1 domains of La proteins are not only implicated in RNA stabilization, but also in RNA folding and RNP assembly (Naeeni et al. 2012; Collopy et al. 2018; O’Connor and Collins 2006; Akiyama et al. 2012). Similarly, AtLa1 might act during the initial steps of telomerase assembly as a chaperone for AtTR. The presence of multiple RNA conformers in our EMSA analyses suggests structural heterogeneity of AtTR, perhaps reflecting alternative folding states that influence protein recognition during RNP assembly. Under these conditions, protein binding is likely coupled to RNA folding, such that AtLa1 preferentially recognizes and stabilizes a binding-competent conformation (conformational selection) and/or promotes its formation through RNA remodeling. Accordingly, the binding affinities derived from our EMSA experiments, where AtTR truncations show multiple bands, should be interpreted as apparent binding constant (*K*_*d,app*_) measured under the defined in vitro folding conditions rather than intrinsic thermodynamic dissociation constants for a single RNA conformation. Although our data support direct binding of AtLa1 to specific structural elements of AtTR, the extent to which AtLa1 stabilizes or remodels AtTR structure during biogenesis remains to be determined. Future structural probing approaches, such as SHAPE or DMS-based analyses performed in the presence and absence of AtLa1, could be useful in revealing RNA conformational changes associated with complex formation.

Taken together, our results provide novel insights into the biogenesis and evolution of plant telomerase. Unlike other eukaryotic telomerases that rely on LARP7 proteins, *Arabidopsis* telomerase seems to have co-opted its genuine La protein AtLa1 for RNP assembly. The requirement for AtLa1 as well as dyskerin not only highlights cooperation of multiple RNA-binding proteins but also implies plants evolved a hybrid strategy for telomerase assembly in which a canonical La protein functions in early RNP biogenesis and is subsequently replaced by other accessory proteins to facilitate optimal enzyme function at telomeres.

## Materials and methods

### Generation of plant transgenic lines and growth conditions

Super-telomerase lines were created with pHSN6A01_U6AtTR_35S_gTERT construct in a *tert* mutant background (Barcenilla et al. 2023). The AtLa1 over-expression line was a generous gift (Cui et al. 2015).

### Inducible AtLa1 knockdown lines

β-Estradiol-inducible RNAi knockdown AtLa1 transformants were created in the wild type (Col-0) background. For plasmid construction, cDNA was synthesized from RNA of Col-0 with SuperScript™ III Reverse Transcriptase. 213 bp of coding region of RRM1 domain of AtLa1 protein was selected for construct preparation. Sense fragments were amplified with X*ho*I and *EcoR*I, and the same antisense fragments were amplified with *BamH*I and *Xba*I restriction digestion sites with the help of primers, cDNA, and Q5 high-fidelity master mix (New England Biolabs). Both sense and antisense fragments were inserted into the hp RNAi pHANNIBAL plasmid by restriction digestion and ligation cloning. A fragment containing senseAtLa1_pdk intron_antisense AtLa1_OCS terminator was inserted into the β-estradiol-inducible XVE destination plasmid pER8 with *Spe*1 and *Xho*I restriction digestion by restriction digestion and ligation cloning.

### Genetic transformation

One-month-old wild type Col-0 plants were transformed by *Agrobacterium*-mediated floral bud transformation with *Agrobacterium* cells (Zhang et al. 2006). Transformed *Agrobacterium* colonies (GV3101) were screened by plating on YEP plates supplemented with 100 µg/ml Rifampicin and 50 µg/ml Kanamycin and colony PCR. Transformed cells were suspended in a buffer (0.002% MS, 5% sucrose, and 0.04% silwet-77). *Arabidopsis* inflorescences were dipped in the solution and vacuum infiltrated for 1.5 min. Treated plants were kept horizontal and covered with plastic film and aluminum foil to retain moisture. After 24 h, plants were transferred to a standard growth chamber. T0 transformants were screened by plating on 0.5 MS supplemented with 30 μg/ml Hygromycin. T2 and T3 plants were screened for a single copy insert and homozygous lines by plating. Transformed stable lines were characterized by western blotting of seven-day-old seedlings grown 0.5 MS plate supplemented with 25 μM β-estradiol. Two stable homozygous T3 transformed inducible RNAi knockdown lines (1–7-6 and 4–3-1) were used for in vivo investigation of telomerase activity investigation. *A. thaliana* plants were transferred in 3:1 Pro-Mix Bio-fungicide and vermiculite: and grown under long-day conditions (16 h light; 8 h dark cycles).

### Quantitative telomere repeat amplification protocol (qTRAP) and telomere repeat amplification protocol (TRAP) assay

Total protein was extracted in buffer W (50 mM Tris–acetate, pH 7.5, 5 mM MgCl_2_, 2 mM Potassium Glutamate, 10 mM EGTA, 1.5% glycerol, v/v) with 1 mM DTT and 0.6 mM VRC. Total protein was measured by Qubit^®^ Protein Assay. Protein samples were diluted to a final concentration of 50 ng/µL. For qPCR, a total 525 ng protein from each sample was added to qPCR plates with 400 µM forward primer and HS dynamo SYBR green master mix for primer extension and plate was kept at 37 °C for 45 min. 400 µM reverse primer was added to the reaction mixture. qPCR was performed and data were analyzed. For the TRAP assay, 525 ng protein was added to reactions containing 1 × primer extension buffer (50 mM Tris-OAc pH 8.0, 50 mM KCl, 3 mM MgCl_2_, 2 mM DTT, 1 mM spermidine), 0.66 µM forward primer (5’ CACTATCGACTACGCGATCAG 3’), 0.83 mM dATP, 0.83 mM dTTP and 0.83 mM dGTP. Reactions were kept at 37 °C for 45 min. The reaction mixture was then subjected to Phenol/Chloroform extraction followed by overnight ethanol precipitation. After centrifugation at 15,000 rpm, 4 °C for 30 min, the resulting pellet was washed with 70% ethanol and re-suspended in nuclease-free water. Re-suspended pellet was used as the template for PCR which was also composed of GoTaq® Hot Start Colorless Master Mix (Promega), 0.4 µM forward primer (5’ CACTATCGACTACGCGATCAG 3’), 0.4 µM reverse primer (5’ CCCTAAACCCTAAACCCTAAA 3’) and 66 nM of [α-32P] dGTP-3000 Ci/mmol (PerkinElmer). After 35 cycles of PCR, the reaction was precipitated and resolved by 6% denaturing PAGE, dried, exposed and imaged on Typhoon FLA 9500 phosphorimager (GE Healthcare).

### qRT-PCR

RNA was extracted from *A. thaliana* 7-day-old seedlings and one- to two-week-old cell culture using the Direct-zol RNA kit (Zymo Research). cDNA was synthesized from 1 µg RNA, SuperScript III reverse transcriptase (Invitrogen), and random primer mix (NEB). cDNA was five-fold diluted and added into PowerUp SyBr Green for qPCR reactions with AtTR primer provided in Supplementary and U6 snRNA is used as a reference for AtTR enrichment.

### Western blotting

Total protein was extracted from 25 mg seedling in a buffer (120 mM Tris base pH 6.8, 4% SDS, 1% Bromophenol blue, 20% glycerol, and 200 mM DTT). Samples were lysed and denatured at 90 °C for 8 min. Samples were centrifuged at 14000 g for 5 min. Equal amounts of samples were analyzed by SDS-PAGE with the marker run at 110 V for 2 h. Protein was transferred from the gel to the activated PVDF membrane by a trans blot turbo system (Bio-Rad) and the membrane blocked in 5% milk. The membrane was incubated in 1:1000 of primary antibody (anti-AtLA1) at 4 °C overnight, and after 24 h, washed thrice in the TBST buffer (TBS and 0.1% Tween 20). RuBisCO acted as a loading control. Finally, the membrane was incubated with 1:20,000 ⍺-rabbit HRP, visualized with an Amersham^™^ Imager 600, and the image analyzed by Image J software.

### RIP

RNA-IP was performed with 1 g of overexpressed and wild type tissue from 7-day-old seedlings. Seedlings were crushed in liquid nitrogen and homogenized in RIP buffer (100 mM Tris–acetate, 150 mM Potassium Glutamate, 1 mM MgCl_2_, 0.5% Triton X-100, O.1% Tween 20) with 2.5 mM DTT, 20 µl/mL protein protease inhibitor (Sigma-Aldrich), and 1 µl/mL RNase OUT (Thermo Fisher Scientific) and centrifuged at 5000 g for 4 min at 4 °C. 1/100th of supernatant was kept as input, and one volume of supernatant was incubated with anti-HA-tagged magnetic beads (Pierce^™^ Anti-HA Magnetics Beads) for 2 h at 4 °C and then washed with RIP buffer four times. qPCR and western blot (probed with anti-HA-HRP) were performed on beads and input.

### Protein expression and purification

AtLa1_WT_ (residues 1-433), AtLa1_ΔC_ (residues 1-397), AtLa1_RRM2_ (residues 298-397), and Dyskerin_ΔC_ (residues 1-439) coding DNAs were cloned into a pET28a vector with an N-terminal 6xHis-SUMO tag. The constructs were transformed into *E. coli* BL-21 DE3 Rosetta cells which were grown overnight at 30 °C. Cells were re-suspended in buffer (500 mM Tris–HCl, 500 mM NaCl, 5 mM imidazole, pH 7.5) and lysed by sonication. Dyskerin_ΔC_ expressing cells were lysed by a micro-fluidizer to obtain a higher amount of protein. The protein extract was purified on an ÄKTA go^™^ protein purification system (Cytiva). HisTrap FF column (5 mL) was used to capture His-tagged proteins. The eluted fractions were assessed for size and purity by 12.5% PAGE. AtLa1_WT_, AtLa1_ΔC_, and AtLa1_RRM2_ fractions showing the expected protein band were subjected to SUMO protease (Sigma/Thermo) digestion, and His-tagged impurities were removed by passing the reaction through a 5 mL HisTrap FF column. The His-SUMO tag on Dyskerin_ΔC_ was not removed. Finally, AtLa1_WT_, AtLa1_ΔC_, and Dyskerin_ΔC_ were purified through Superdex^®^ 200 Increase 10/300 and AtLa1_RRM2_ was purified through Superdex^®^ 75 10/300 GL gel filtration columns (GE Healthcare) in a buffer (50 mM Tris–Cl pH 7.3, 350 mM KCl and 2 mM DTT). Protein fractions were stored at -80 °C until further use.

### Affinity purification of TERT for mass spectrometry

5 g *Arabidopsis* flowers from super-telomerase plants or *tert* mutant plants (untransformed control) were ground in liquid nitrogen and homogenized in 20 ml lysis buffer (100 mM Tris-OAc pH 7.5, 150 mM KGlu, 5 mM MgCl_2_, 0.1% Triton X-100, 20 mM EGTA, 15 g/L PVP, 10% glycerol, 20 μl/ml plant protease inhibitor mixture [Sigma-Aldrich], 1 μl/ml RNaseOUT [Thermo Fisher Scientific], and 2 mM DTT). The extract was centrifuged at 4℃, 13,000 rpm (> 14500 g) for 15 min, and the supernatant was collected. A Strep-Tactin XT 4 flow gravity column (0.2 ml) high capacity (iba: 2–5031-005) was equilibrated with 5 column volumes of equilibration buffer (100 mM Tris–OAc pH 7.5, 150 mM KGlu, 5 mM MgCl_2_, 0.1% Triton X-100, 10% glycerol, and 2 mM DTT). The supernatant was loaded onto a Strep-Tactin^®^XT 4Flow^®^ high-capacity column (IBA Lifesciences GmbH) at 4 °C for 1 h. The column was washed with 2.5 ml wash buffer 1 (100 mM Tris-OAc pH 7.5, 150 mM KGlu, 5 mM MgCl_2_, 0.1% Triton X-100, 10% glycerol, 20 μL/mL plant protease inhibitor mixture [Sigma-Aldrich], 1 μl/ml RNaseOUT [Thermo Fisher Scientific], and 2 mM DTT). Samples were then washed with 2 mL wash buffer 2 (100 mM Tris–OAc pH 7.5, 150 mM KGlu, 5 mM MgCl_2_, 10% glycerol, 20 μl/ml plant protease inhibitor mixture, 1 μl/ml RNaseOUT, and 2 mM DTT). Finally, the column was washed with 0.6 ml wash buffer 3 (100 mM Tris–OAc pH 7.5, 150 mM KGlu, 5 mM MgCl_2_, 10% glycerol, and 2 mM DTT). The captured proteins were eluted with 8-column volumes of 20 mM NaOH (Strep-Tactin XT 4 Flow). 1 M Bis–Tris–HCl, pH 5.8, was added to each eluted fraction to take the pH to 7.0–7.5 (final Bis–Tris concentration 30 mM). Eluted products were concentrated to 50–100 μl by a 5 kDa concentrator. (Amicon^®^).

### Quantitative mass spectrometry

50 µl of TFE (trifluoroethanol) was added to 50 ml affinity-purified protein sample, and proteins were reduced, alkylated, and digested with trypsin as in Blank et al. 2020 (PMID 32129706). Digests were filtered (10 kDa Amicon Ultra, Millipor # UFC5010BK) prior to desalting on C18 (Thermo Scientific Hypersep SpinTip # 60,109–412). Peptides were dried, re-suspended in 40 µl 5% acetonitrile/0.1% formic acid for LC/MS–MS using reverse phase chromatography on a Dionex Ultimate 3000 RSLC nano-UHPLC system (Thermo Scientific) with a C18 trap to Acclaim C18 PepMap RSLC column (Dionex; Thermo Scientific) configuration. Peptides were eluted using a 3% to 40% gradient over 60 min with direct injection into a Thermo Orbitrap Fusion or Fusion Lumos Tribrid mass spectrometer using nano-electrospray. Data were collected using a data-dependent top-speed HCD acquisition method with full precursor ion scans (MS1) at 120,000 m/z resolution. Monoisotopic precursor selection and charge-state screening were enabled using Advanced Peak Determination (APD), with ions of charge 2–6 selected for high energy-induced dissociation (HCD) with stepped collision energy of 30% ± 3%. Dynamic exclusion was active for ions selected once with an exclusion period of 20 s. All MS2 scans were centroid and collected in rapid mode. For each biological replicate, data were collected for 1–3 injections. Raw MS/MS spectra were processed using Proteome Discoverer (v2.5) using the 2024 UniProt *Arabidopsis thaliana* reference proteome, UP000006548, a common contaminants fasta, and a PWF_Tribrid_Basic_Percolator_SequestHT workflow. Results were filtered for high-confidence proteins (FDR 1%) with at least 2 peptide spectral matches (PSMs) and contaminant proteins were removed prior to statistical analysis using the edgeR algorithm implemented in Degust (https://doi.org/10.5281/zenodo.3258932). We required a minimum PSM count of 2 in at least 2 samples and implemented the RUVr edgeR-quasi-likelihood model with TMM normalization. Mass spectrometry data are deposited in the ProteomeXchange repository (#PXD071051).

### EMSA

Telomerase RNA, its truncations, and variant RNAs were generated with an AmpliScribe^™^ T7 High Yield Transcription Kit and purified by 6% denaturing PAGE. Purified RNAs were radiolabeled at the 5’ end with ^32^P-ATP (Revvity) using T4 Polynucleotide kinase (NEB) and PAGE-purified. [^32^P] labeled RNAs were folded by heating at 98 °C for 2 min and slow-cooling to RT. Labeled RNAs were added to the EMSA reaction buffer (25 mM Tris–Cl pH 7.5, 50 mM KCl, 2 mM MgCl_2_, 10% glycerol, 1 mM DTT, 50 µg/ml BSA, 50 µg/ml yeast tRNA, and 1 µl/ 20 µl RNaseOUT [Thermo Fisher Scientific] and were incubated at 30 °C for 10 min. AtLa1_WT_ was serially diluted, mixed with the RNA-containing EMSA reaction, and incubated at 30 °C for 20 min. EMSA products were separated on 6% native PAGE in TBE buffer at 4 °C. Gels were dried under vacuum at 80 °C for 1 h before exposure to a phosphor imager screen. Data were collected on Typhoon FLA 9500 phosphorimager (GE Healthcare) and quantified using ImageJ. The fraction of RNA bound at each protein concentration was calculated as the ratio of bound RNA to total RNA (bound + unbound). Due to the presence of multiple stable RNA conformers on native gels, quantification using ImageJ was restricted to the primary binding-competent free RNA species (the active fraction). Binding curves were fitted using a specific binding model with Hill slope in GraphPad Prism to calculate dissociation constants (*K*_*d*_) or apparent dissociation constants (*K*_*d,app*_).

### In vitro telomerase reconstitution

An in vitro telomerase reconstitution assay was performed as described previously (Song et al. 2021).

### Telomere length analysis

Telomere length measured by Primer Extension Telomere Length Analysis (PETRA) assay and mean telomere length analyzed by an online tool WALTER analysis (Lyčka et al. 2021). PETRA and WALTER analysis followed a well-established protocol from Barcenilla et al. 2023.

### Statistical analysis

Statistical analysis was performed by GraphPad Prism 9 from MacOS and analysis was considered. We used more than three biological replicates for qTRAP analysis and the results were considered statistically significant when the *p*-value was lower than 0.05.

## Supplementary Information

Below is the link to the electronic supplementary material.

Fig. S1. Over-expression of AtLa1 does not alter telomere length. (A-B) Characterization of over-expression tagged AtLa1 by western blot probed with anti-HA-HRP. Molecular weight markers are (37-75 kDa) shown on the left of blots. (C) Western blotting results for tagged and endogenous AtLa1 protein expression in transformed lines OE AtLa1-2, OE AtLa1-3,OE AtLa1-10 as compared to wild type Col-0. Blots were probed with α-AtLa1 primary antibody. (D-F) Telomere length distribution in over-expression AtLa1 lines and wild type of Col-0 as measured by Primer Extension Telomere Length Analysis (PETRA) in seven-day-old seedlings. Results are shown for the telomeres on chromosome arms 1L, 2R, and 5R (TIF 654 kb)

Fig. S2. Transient knockdown on AtLa1 does not alter telomere length. (A) Results of Quantitative Telomere Repeat Amplification Protocol assays (qTRAP) for25 µM β-estradiol incubated wild type Col-0 as compared to untreated wild type Col-0. (B-D) Results of Primer Extension Telomere Length Analysis (PETRA) for telomere length distribution in seven-day-old seedling transformed an AtLa1 inducible RNAi knockdown construct (1-7-6 and 4-3-1) relative to wild type (Col-0). Plants were grown in MS plates supplemented with 25 µM β-estradiol. Results are shown for the telomeres on chromosome arms 1L, 2R, and 5R (TIF 485 kb)

Fig. S3. Purification of AtTR, AtLa1_WT_, AtLa1_ΔC_ and AtLa1_RRM2_. (A) Purification of AtTR using a 6% denaturing polyacrylamide gel (8M urea). The RNA band was visualized by UV shadowing, excised and eluted for subsequent experiments. (B-D) SDS-PAGE analysis of collected fractions of recombinant (B) AtLa1_WT_, (C) AtLa1_ΔC_, and (D) AtLa1_RRM2_. AtLa1_WT_ and AtLa1_ΔC_ were purified through Superdex® 200 Increase 10/300 and AtLa1_RRM2_ was purified through Superdex® 75 10/300 GL gel filtration columns (TIF 1170 kb)

Fig. S4. C-terminal of AtLa1 is critical for binding with AtTR. EMSAs with AtTR truncation A reveal that AtTR cannot interact with (A) AtLa1_ΔC_ lacking c-terminal and (B) AtLa1_RRM2_, which is conserved among LARP7 proteins (TIF 670 kb)

Table S1. Mass spectrometry protein identification (XLSX 695 kb)

## Data Availability

The data that support the findings of this study are available from the corresponding author upon reasonable request.
